# Individual and Group Psycho-Educational Counseling on Knowledge, Attitude and Preference for Birth Method in Nulliparous Women: A Randomized Controlled Trial 

**Published:** 2018-06

**Authors:** Mahnaz Nosratabadi, Khadija Hekmat, Anna Dencker, Zahra Abbaspoor

**Affiliations:** 1 Department of Midwifery, Faculty of Nursing and Midwifery, Dezful University of Medical Sciences, Dezful, Iran; 2 Department of Midwifery, Faculty of Nursing and Midwifery, Ahvaz Jundishapur University of Medical Sciences, Ahvaz, Iran; 3 Reproductive and Perinatal Health, Centre for Person-Centered Care, Institute of Health and Care Sciences, Sahlgrenska Academy, University of Gothenburg, Gothenburg, Sweden; 4 Department of Midwifery, Reproductive Health Promotion Research Center, Ahvaz Jundishapur University of Medical Sciences, Ahvaz, Iran

**Keywords:** Counseling, Knowledge, Attitude, Birth Method Selection

## Abstract

**Objective:** To study the effects of the Individual and Group Psycho-educational counseling in pregnant women on knowledge, attitude and mode of delivery.

**Materials and methods:** This is a randomized controlled trial that carried out on 100 healthy nulliparous pregnant women with uncomplicated pregnancies, who had no contraindication for vaginal birth, but opted for a caesarean section in Medical centers of Dezful city, in the south west of Iran. Participants were randomly assigned into individual or group psycho-educational counseling from gestational week 20 and Knowledge, attitude and mode of delivery in the Individual and Group Psycho-educational counseling methods were measured.

**Results:** All the participants (100%) in the individual and a majority (92%) in the group counseling changed their preference for birth method to vaginal birth after the counseling intervention. Baseline mean scores of knowledge and attitude into birth method selection were equal between groups. After the counseling intervention the mean scores increased significantly for knowledge in both the individual and group counseling groups: 12.96 and 12.88 before the intervention to 24.16 and 22.62, respectively (p < 0.001). Likewise attitude mean scores increased in both groups: 116.06 and 123.42, respectively, before the counseling sessions, that changed to 170.12 and 160.36 after the counseling sessions (p < 0.001). The differences in knowledge and attitude mean scores were statistically non-significant between groups after the intervention.

**Conclusion:** The individual as well as the group psycho-educational counseling sessions increased the knowledge and attitude of pregnant women in relation to vaginal birth without significant differences between groups. Both methods can be recommended. A group counseling method is more effective for advising on the choice of delivery method when many women request a caesarean section without medical indications.

## Introduction

The childbirth is considered as an important experience in women^'^s life (1) and is deemed to have profound effects on mental and social health of women and their families (2). Normal vaginal birth is a spontaneous process without any intervention, except when complications occur (3).

Caesarean section*;* When complications threatening mother or the fetus, such as fetal asphyxia, placenta abruption, or events that interrupt the normal birth process, cesarean section (CS) could be considered (4). CS is associated with complications, including hemorrhage, blood transfusion, hysterectomy, the risk of damage to other organs, subsequent complications, prevalence of infertility, ectopic pregnancy (5-7) and postpartum depression  (8). The risk of maternal mortality is 7 times higher in CS than a spontaneous vaginal birth (9).

The overall rate of CS in a country is an indicator of performance evaluation of maternal health programs and in accordance with the recommendations of the World Health Organization, the CS rate should not be more than 15% (10). Statistics show that in recent years the overall trend of CS in Iran has increased more than the global standard putting Iran in the second place after Brazil in terms of the CS rate. Also, Iran ranked first in the CS rate among the Middle East countries. For a country like Iran, which has been successful in promoting a variety of health indicators, the present ranking is a matter of concern (11). The increase in the CS rate in Iran may reflect a poor performance of the health system (12). Furthermore, several studies have shown that 75% of the CSs in Iran were unnecessary and opted for by the mothers (13).

***Women's knowledge and attitudes of birth method:*** Several factors are involved in the growing CS rate, including fear of childbirth  (14), fear of lack of control over the childbearing events, fear of pain, the mother's choice, and the lack of knowledge regarding the CS disadvantages and vaginal birth advantages as well as women’s negative attitudes towards their previous vaginal delivery (15). Also, the psychological factors such as fear, depression and anxiety are believed to have significant roles in birth method selection (16).

On the other hand, attitude is highly associated with awareness. So, for any change in attitude, providing an appropriate context to raise awareness is important (17), Also, lack of awareness of the complications of CS, negative attitude, rumors and false of postnatal complications about vaginal birth, and also advertisements for CS are important reasons that cause women opt for CS (18), So, by promoting the knowledge and attitude of women toward vaginal birth, their motivation is to go for vaginal birth is increased. One of the effective strategies to help women in this regard is the counseling and training of the pregnant women.

***Psycho-educational counseling during pregnancy: ***Counseling, as an interaction between counselor and client, is expected to lead to changes in one or all domains of behavior, personal characteristics, and also the decision-making process. The psycho-educational counseling as well as health promotion and education are deemed to involve all three levels of prevention. In face to face counseling, by providing a private situation, focusing on the individual characteristics of the clients, they will be assisted to know themselves and then make a reasonable and acceptable decision to develop and discover solutions for their behavioral problems. In fact, face to face counseling is done by considering the learning capacity and understanding of clients in a wide variety of therapeutic intervention methods (19). The other effective method in counseling is the group counseling. It is considered as a series of activities conducted with a certain number of participants at the same time. These activities have curative and preventive aspects (20). A recent investigation has shown that the psycho-educational group counseling could change the selected birth method in women who chose CS. Besides, group counseling was reported to help women in compliance with the stages of pregnancy and childbearing preparing them for childbearing with a positive attitude  (14).Also, counseling during pregnancy could increase the rate of vaginal birth, a better first-minute Apgar score, increasing the newborn weight and reducing the length of stay in the neonatal unit (21). In another study, individually counseling to primiparous women in the third trimester of pregnancy was reported to be effective in reducing the rate of anxiety during labor and childbearing, and also in increasing the vaginal delivery (22).

Most of the counseling and training with regard to childbearing have been done as group counseling, and only a few studies has focused on the efficacy of individual counseling. Therefore, the present study aimed to compare the effect of individual and group psycho-educational counseling sessions on knowledge, attitude and preference formode of delivery in pregnant nulliparous women.

## Materials and methods

This randomized controlled trial was carried out in 6 health centers in Dezful city in the south west of Iran from April to October 2016. Data collection and counseling sessions were held by the first author who is a certified midwife (MN).With 90% power and an alpha level of 0.05 in a two-sided test, and according to the study of Abedian et al. results (2012) (23), the sample size was calculated to be 45 for each group. Assuming that attrition will be less than 10%, 50 women were needed in each randomized group.

The group allocation was done through computer generated randomized sequencing. 

**Figure 1:**
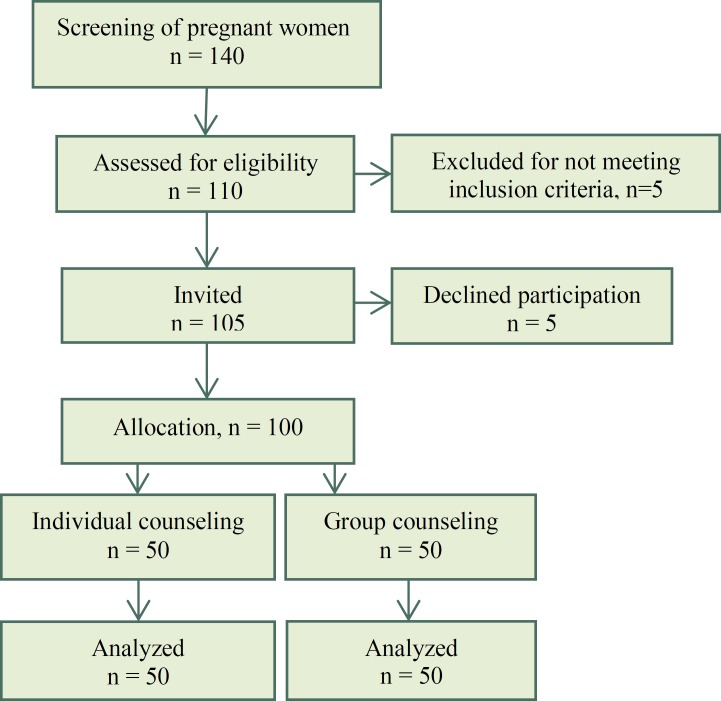
Flow diagram of the recruitment and retention of participants in the study

Inclusion criteria were nulliparous women with uncomplicated pregnancy, singleton pregnancy, gestational age between 20 to 26 weeks, literacy of mothers, and requesting an elective caesarean section (SC) for other than medical reasons. Exclusion criteria were high risk pregnancy (preeclampsia, diabetes, uncontrolled gestational diabetes, previous pre-term labor, previous intrauterine fetal death and so on), having a history of infertility, contraindication for normal vaginal birth, no tendency for the mother or her husband to participate in the counseling sessions. In total 140 women were screened for inclusion, 110 were assessed for eligibility, 105 fulfilled inclusion criteria and were informed of the study. Five women declined participation and 100 women gave written, informed consent to participate (Figure 1).

At first stage 140 pregnant women were asked to participate in the screening phase of the trial and 110 women had a request for cesarean section. Out of 110 women 5 women refused to participate, and 5 women not meeting inclusion criteria and 100 women entered the study, then they were divided randomly into two individual counseling group and group counseling group (50 women in each group) using a random draw method.

At inclusion two groups were asked to fill in a questionnaire about socio-demographic questionnaire including personal, social and reproductive questions. Participants also answered the knowledge and attitude questionnaires concerning the preferred birth method (24). The first researcher (MN) was responsible to perform the counselling and follow-up participants after recruitment. All sessions were held in the health centers.

All participant received counselling individually or in groups of ten women. The individual and group counseling groups received the same counseling method in 8 sessions during 8 weeks, but in individual group the duration of counseling was 45-60 minutes, and in group counseling group the session was lasted 90-120 minutes for every 10-person group. Based on the clients’ needs, educational tools such as movies and books were used, and they were also served with milk and cake at the end of each session. 

Eight weeks after intervention and at the end of the counseling sessions, the post-test was used to assess clients’ knowledge, attitude, and the elected birth method again. Data for mode of delivery (birth mode) was retrieved from hospital records. At the 6^th^ and 8^th^ meeting, husbands could accompany their wives in attendance**.**

The content of the counseling sessions was provided based on the counseling skills and health counseling (25) using micro skills technics of Rajerss counseling (26), and the counseling sessions were administered by the first researcher as a midwifery counselor.

1-2) During the first counseling sessionthe objectives of the counseling sessions were explained, and members in the two groups got familiar with the procedure of the sessions. Besides, an open and trusting relationship was established, and it was assured that client information and problems were confidential. In the second session, information regarding the clients’ needs, anxieties and concerns were obtained. After the second sessions, 30 last minutes of every session were dedicated to relaxation. 

3) In the third session, the client attitude, perceptions and beliefs about the current issues related to the birth methods were identified. 

4) In the fourth session of counseling, causes of fear and anxiety for women with regards to normal birth were investigated, and due consultation was provided for solving them.

5-6) at the fifth session, clients’ awareness about the advantages and disadvantages of each mode of birth was sought for. To familiarize the clients with issues such as hospital routine and the process of labor, pain relief techniques, and bodily changes in the postpartum period, becoming a mother, identifying and understanding the symptoms of postpartum depression and bonding with the baby, the sixth session was run. In this session the clients were accompanied by their husbands in the both groups.

7) In the seventh session the advantages and disadvantages of different methods of delivery and their impacts on the mother and baby were explained. 

8) In the eighth session, the clients and their husbands attended the counseling session again in the both groups. At this meeting, the process of becoming a family, changes in relationships, parenthood, improving mutual understanding between parents, baby/neonate bonding, and also planning for birth were introduced (14, 25, 26).

Validity and reliability of the knowledge and attitude questionnaires was approved by Akbari et al, 2007. The knowledge questionnaire (consisted of 25 questions with 4 options: a correct answer rendered a score of 1, while a wrong or no answer gave a score of 0). The attitude (consisted of 40 questions based on a Likert scale (strongly agree, agree, neutral, disagree and strongly disagree), assessed attitude of the pregnant women about their mode of delivery (26). The response options “strongly agree” and “agree” were considered as a positive attitude, and “disagree” and “strongly disagree” were considered as a negative attitude. 

The mean scores on the knowledge and attitude about birth methods questionnaires, before and after the intervention were compared between the randomized groups. The Mann–Whitney U test was used to compare means. Proportions of events were compared with independent t-test or chi-square analysis. For parametric data, the t test for two independent groups was used, while for non-normally distributed data, the Mann–Whitney U-test was applied. Data were analyzed using SPSS software (version 22). A p-value less than 0.05 was considered significant.

Ethical issues of the study were approved by the research ethics committee of Ahvaz Jundishapur University of Medical Sciences, Ahvaz, Iran (IR.AJUMS.REC.1394.92). In addition, informed consent (oral and written) of all participants was obtained. This study was registered with the registration code IRCT2015061422720N1 in the clinical trial website.

## Results

During the study period, 100 women preferring elective CS were randomized to individual or group psycho-educational counselling groups. There were no differences in socio-demographic characteristics between the randomized groups (Table 1).

Forty-six women (92%) from the group counseling and all participants (100%) in the individual counseling group changed their preference for elective caesarean section and wanted a vaginal birth after the counselling sessions. 

Also, the results showed that based on Chi-square test, there is no significant difference between the group and individual counseling groups in terms of knowledge (p = 0.42) and attitude (p = 0.49), but within the individual counseling group, a moderate level of knowledge of birth methods elevated from 56% before the counseling sessions to 100% after attending them. Also in the group counseling, the moderate level of knowledge of birth methods elevated from 68% before the counseling sessions to 96%, a good level of knowledge, after attending them (p < 0.001).

Likewise, in terms of attitude there was no significant difference between the groups (p = 0.49). Moreover, in the evaluation of the attitude level of the participants on birth methods in the individual counseling group, before the intervention, 12% had positive and 88% had a neutral attitude, while after completing the counseling sessions, 100% of the participants changed their attitude to positive. Also, in the group counseling, before the intervention, 20% of the participants had a positive and 80% of them had a neutral attitude, while it changed to 96% positive and 4% neutral attitude at the end of the counseling sessions.Each group experienced a statistically significant change in terms of attitude after the intervention (p < 0.001) (Table 2).

**Table 1:** Socio-demographic characteristics of participants in group and individual counseling groups

**Variables**	**Individual counseling ** **n = 50**	**Group counseling** **n = 50**	**p-value**
Age, y, Mean (SD) (years)	28.7 ±4.2	26.4 ± 3.12	0.756
Husband Age, y, Mean (SD)	30.58 ± 4.01	30.84 ± 4.04	0.785
Ethnicity, n (%)			
Persian	39 (78)	43 (84)	0.693
Lor	9 (18)	8 (16)	
Other	2 (4)	0 (0)	
Education, n (%)			
Diploma and under the diploma	15 (30%)	16 (32%)	0.99
University	35 (70%)	34 (68%)	
Husband Education, n (%)			
Diploma and under the diploma	18 (36%)	22 (44%)	0.54
University	31 (62%)	28 (56%)	
Job, n (%)			
Housewife	45 (90)	49 (98)	0.20
Employee	5 (10)	1 (2)	
Family income level, n (%)			
Very good	0 (0)	4 (8)	0.08
Good	18 (36)	17 (34)	
Moderate	31 (62)	25 (50)	
Weak	1 (2)	4 (8)	
Housing situation, n (%)			
Owner	25 (50)	19 (38)	0.42
Rental	13 (26)	15 (30)	
Living with husband family	12 (24)	16 (32)	
Insurance, n (%)			
Yes	48 (96)	47 (94)	0.99
No	2 (4)	3 (6)	

In terms of opting for a birth method, in the individual counseling group, 78% had a successful vaginal birth, 14% had an emergency CS, and 8%, on the advice of the doctor in the admission time, gave birth through CS. In the group counseling 70% had a successful vaginal birth, 8% had an elective CS, and 12% had an emergency CS, while 10% performed CS according to doctor's advice in the admission time to hospital. Overall, there were no statistically significant differences between the two groups (Table 3).

## Discussion

The results showed that educational-psychological counseling in the individual as well as the group counseling method could change the preference from elective caesarean to vaginal birth. Among women who preferred vaginal birth, most of women in the both groupsalso gave birth vaginally. These results are in line with the findings of Malakouti et al. study that an educational intervention increased the knowledge of pregnant women (27). Likewise, another research showed that the effect of education on birth method selection increased the awareness of women with regards to the birth methods, and the rate of CS decreased in the educated group significantly (28). Also, in the study conducted by Saisto et al. (2007), the positive impact of psychological practices in reducing anxiety during labor was reported (29). Artita et al. (2010) reported that mothers in training methods, learn the problem-solving approaches and deal with childbirth as a solvable problem (30). These results for the group counseling are in line with the results of Sanavi et al. (2014) study that the education based on planned behavior therapy on birth method selection was remarkably associated with the positive attitude toward normal vaginal birth (31). Moreover, in Malakouti et al. study (2014), group education as lectures and power point presentations during four 90-minute sessions per week increased the positive attitude of nulliparous women toward normal vaginal birth (27).

**Table 2:** The knowledge and attitude levels about birth methods, before and after intervention in group and individual counseling groups

**Variables**			**Individual counseling ** **n = 50** ***N(%)***	**Group counseling** **n = 50** ***N(%)***	**p-value** **between ** **groups**
Knowledge					
Pre-intervention		Good	14 (28)	9 (18)	0.42
		Moderate	28 (56)	4(68)	
		Weak	8 (16)	7 (14)	
Post intervention		Good	50 (100)	48 (96)	0.49
		Moderate	0 (0)	2(4)	
		Weak	0 (0)	0 (0)	
P-value (pre and post)			0.001	0.001	
Attitude					
Pre intervention	Positive		6 (12)	10 (20)	0.27
	Neutrals		44 (88)	40(80)	
	Negative		0 (0)	0 (0)	
Post intervention	Positive		50 (100)	48 (96)	0.49
	Neutrals		0 (0)	2(4)	
	Negative		0 (0)	0 (0)	
P-value (pre and post)			0.001	0.001	

The results of this study also showed that both groups had an increased number of selected normal vaginal births. Saisto and Halmesmäki (2007), after implementing psychological counseling and relaxation training, reported that most pregnant women who had fear of childbirth had a normal vaginal birth (29).The results of this study in the selection of birth method are also in accordance with Khan-Jeihooni et al. and Rouhe et al. studies (14, 32). However, our findings are not in line with the Sydsjö et al. (2014) findings that individual counseling in women who had a fear of labor was not effective, and there were less vaginal births and more requests for CS. This lack of alignment can be due to different types of intervention methods, such as the difference between the number of counseling sessions, consultation conditions of counselors, and their study method that was a descriptive, retrospective cohort and case-control study (33).

**Table 3:** The frequency of birth methods selected, before and after the intervention in group and individual counseling groups and mode of delivery

**Variables**		**Individual counseling ** **n = 50** ***N (%)***		**Group counseling** **n = 50** ***N (%)***	**p-value**
Pre-intervention					
Cesarean section		50 (100)		50 (100)	0.11
Normal vaginal delivery		0 (0)		0 (0)	
Post intervention					
Cesarean section		0 (0)		4 (8)	
Normal vaginal delivery		50 (100)		46 (92)	
P-value (pre and post)		0.001		0.001	
Birth methods selected					
Selected Cesarean section	0 (0)		4 (8)		0.26
Vaginal delivery	39 (78)		35 (70)		
Emergency CS	7 (14)		6 (12)		
Advised CS by physician	4 (8)		5 (10)		

Noteworthy in the present study was a significant change that happened after the intervention in choosing the preferred birth method. In effect, the participants in this study all wanted CS first and were not willing to go for vaginal birth method; however, in the individual psycho-educational counseling group as same as group counseling group, after attending the counseling sessions, they became interested in normal vaginal birth.

## Conclusion

The results showed that there were no differences between the individual and group psycho-educational counseling methods about the preferences for elective CS or vaginal birth. In both groups, psycho-educational counseling method was found to be an effective method to raise awareness level and positive attitude of vaginal birth in nulliparous pregnant women. Therefore, this method could help women to make an informed decision regarding preference for birth method. The use of this method is likely to increase the rate of normal vaginal birth and reduce the rate of CS health care costs when many women opt for an elective CS. Since this method of consultation was conducted in Iran for the first time, it is suggested that a broader and more diversified geographical distribution be considered in future studies. In addition, with regard to the efficacy of these types of counseling, it suggested that according to the number of clients, both counseling methods could be used in antenatal care centers for women who request a CS with no medical indication.
